# Inital experience of imaging cardiac sarcoidosis using hybrid PET-MR - a technologist's case study

**DOI:** 10.1186/1532-429X-15-S1-T1

**Published:** 2013-01-30

**Authors:** Celia O'Meara, Leon J Menezes, Steven K White, Eleanor Wicks, Perry Elliott

**Affiliations:** 1 Institute of Nuclear Medicine, University College London Hospitals, London, UK; 2 Heart Imaging Centre, Heart Hospital, UCLH, London, UK

## Background

Cardiac imaging has been identified as a potential use of hybrid PET-MRI. This new technology allows simultaneous Positron Emission Tomography (PET) and MR scanning to occur.

This case study features a 34 year old male with known pulmonary sarcoidosis which was diagnosed in 2006 and initially treated with immunosuppressants. He presented with NYHA functional class 2 symptoms. ECHO showed asymmetrical LV anterior and lateral wall hypertrophy with a maximum wall thickness of 18mm in the basal and mid anterior and lateral wall.

The patient was referred for a cardiac18F Fluorodeoxyglucose (FDG) PET-MR study to determine the cause of the hypertrophy. The possible differential diagnoses included hypertrophic cardiac myopathy with pulmonary sarcoidosis or cardiac sarcoidosis.

## Methods

Prior to the scan, the patient followed a high protein, low carbohydrate diet for one day. This was then followed by a 12 hour fast to suppress physiologic glucose metabolism by the heart.

The patient was administered with 355mBq of 18F FDG 180 minutes prior to the PET-MR study.

A simultaneous PET-MR study was acquired. A standard CMR protocol was undertaken in conjunction with a simultaneous 10 minute single PET bed using a Siemens Biograph mMR hybrid PET-MR scanner. This is a 3T magnet containing a 260mm field of view PET detector located at the isocentre.

## Results

The scan demonstrated mediastinal 18F FDG avid lymphadenopathy with anterio-lateral myocardial thickening with mid-myocardial LGE and focal 18F FDG uptake compatible with active myocardial inflammation within an area of "scar" in keeping with active cardiac sarcoidosis.

The oedema sensitive STIR sequence did not differentiate between the hyper-metabolic active disease and the chronic "burnt out" disease.

## Conclusions

This patient is one of the first examples of imaging active cardiac sarcoidosis using a hybrid PET-MR technique. It demonstrates the potential for differentiating between active and chronic cardiac sarcoidosis during one scan. This new imaging technique could have a substantial impact on the diagnosis and management of cardiac sarcoidosis

## Funding

UCL\UCLH receives a proportion of funding from the Department of Health's NIHR Biomedical Research Centres funding scheme.

**Figure 1:**
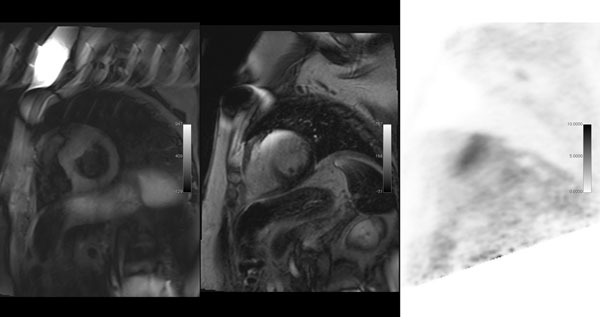
From left to right: 1) SA STIR demonstrating shows homogeneous signal from the LV myocardium. 2) SA LGE demonstrating focal mid myocardial wall LGE in the antero-septal segments. 3) SA 18F FDG PET showing focal uptake in the antero-septal segements Note is made of a Reveal device visualsied on images 1 & 2.

**Figure 2:**
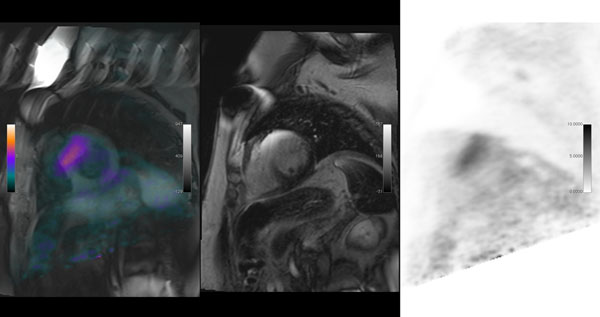
These images show the fusion of the PET onto the STIR sequence, demonstrating the area of active inflammation despite there being no oedema.

